# The Role of Noncoding RNAs in the Regulation of Anoikis and Anchorage-Independent Growth in Cancer

**DOI:** 10.3390/ijms22020627

**Published:** 2021-01-10

**Authors:** Han Yeoung Lee, Seung Wan Son, Sokviseth Moeng, Soo Young Choi, Jong Kook Park

**Affiliations:** Department of Biomedical Science and Research Institute for Bioscience & Biotechnology, Hallym University, Chunchon 24252, Korea; gksdudsd@gmail.com (H.Y.L.); miyanae@naver.com (S.W.S.); sokvisethmoeng@yahoo.com (S.M.); sychoi@hallym.ac.kr (S.Y.C.)

**Keywords:** anoikis, anchorage-independent growth, metastasis, apoptosis, noncoding RNA, microRNA, long noncoding RNA, cancer

## Abstract

Cancer is a global health concern, and the prognosis of patients with cancer is associated with metastasis. Multistep processes are involved in cancer metastasis. Accumulating evidence has shown that cancer cells acquire the capacity of anoikis resistance and anchorage-independent cell growth, which are critical prerequisite features of metastatic cancer cells. Multiple cellular factors and events, such as apoptosis, survival factors, cell cycle, EMT, stemness, autophagy, and integrins influence the anoikis resistance and anchorage-independent cell growth in cancer. Noncoding RNAs (ncRNAs), such as microRNAs (miRNAs) and long noncoding RNAs (lncRNAs), are dysregulated in cancer. They regulate cellular signaling pathways and events, eventually contributing to cancer aggressiveness. This review presents the role of miRNAs and lncRNAs in modulating anoikis resistance and anchorage-independent cell growth. We also discuss the feasibility of ncRNA-based therapy and the natural features of ncRNAs that need to be contemplated for more beneficial therapeutic strategies against cancer.

## 1. Introduction

Anoikis, a specific type of apoptotic cell death, can be instigated when cells lose their interaction with the neighboring extracellular matrix (ECM) [1,2,3]. Failing to receive correct signals from ECM makes detached and misplaced cells unable to proliferate and survive in an unsuitable location, contributing to the maintenance of tissue homeostasis under physiological conditions. For example, loss of integrin-mediated survival signaling upon detachment of cells from ECM activates BCL-2-modifying factor (BMF), giving rise to anoikis [4]. During mammary ductal morphogenesis, anoikis stimulated by BCL2-like 11 (BCL2L11, also called BIM) eliminates cells that are misplaced from adjacent ECM to maintain tissue homeostasis [5].

However, cancer cells can develop resistance toward anoikis, enabling them to survive while spreading from their primary sites to distant organs and to grow under anchorage-independent conditions for the establishment of metastatic lesions [1,2,3,6]. Anchorage-independent growth is a distinctive feature of anoikis-resistant cells and represents the metastatic capacity of cancer cells [2]. In addition, anoikis-resistant cancer cells display high expression of vascular endothelial growth factor A (VEGFA) and facilitate angiogenesis compared to their parental cells [7], supporting that the acquisition of anoikis resistance is a critical prerequisite event for metastasis.

Biological factors underlying anoikis resistance and anchorage-independent cell growth include cellular molecules and signaling pathways linked to apoptosis, cell survival, and cell cycle. For example, anoikis can be facilitated by the activation of BCL2-associated X protein (BAX) and caspase-3 by BCL2L11 [8]. Further, neurotrophic receptor tyrosine kinase 2 (NTRK2, also known as TrkB) suppresses anoikis by activating phosphoinositide 3-kinase (PI3K)/Akt signaling [6]. Besides, anoikis and anchorage-independent cell growth can be inhibited and activated, respectively, by cyclin D1 (CCND1) in cancer cells [9].

Other factors responsible for anoikis resistance and anchorage-independent cell growth are epithelial-mesenchymal transition (EMT), cancer stemness, autophagy, and integrins as well as their downstream mediators such as focal adhesion kinase (FAK, also called protein tyrosine kinase 2 (PTK2)). Anoikis resistance is a hallmark of EMT, and anchorage-independent cell growth can be repressed by cadherin 1 (CDH1, also named E-cadherin) [2,10]. It was also demonstrated that cancer stem cells (CSCs) are resistant to anoikis and also protect non-CSCs from anoikis via the secretion of exosomes [11]. Additionally, autophagy confers anoikis resistance in cancer cells. Treatment with rapamycin (autophagy inducer) and hydroxychloroquine (autophagy inhibitor) blocks and activates anoikis, respectively. Also, knockdown of Beclin-1 (BECN1), a mediator of autophagy, attenuates anoikis resistance, thereby inhibiting the spheroid formation of cancer cells under anchorage-independent conditions [12,13]. Furthermore, FAK mediates integrin signaling and suppresses detachment-induced cell death, supporting anchorage-independent cell growth and metastasis [14]. Inhibition of FAK using genetic and pharmacological approaches weakens anoikis resistance as well as metastasis [15,16].

The degradation and translation repression of target gene messenger RNAs (mRNAs) are commonly regulated by microRNAs (miRNAs). Recently, it was also proposed that the translation of target genes can be enhanced by miRNAs that are bound to G-rich RNA sequence binding factor 1 (GRSF1) [17]. Also, long noncoding RNAs (lncRNAs) are able to serve as molecular sponges, thereby affecting the activity of miRNAs. Besides, lncRNAs have been identified to regulate several events, such as RNA splicing and protein degradation [18,19]. Evidence provided indicated that noncoding RNAs (ncRNAs) regulate anoikis in normal cells. For example, miR-181a-5p can promote anoikis via repressing autophagy in normal mammary epithelial cells [20]. Besides, both miRNAs and lncRNAs can be differently expressed and modulate signaling pathways involved in multitudinous events, such as apoptotic cell death and metastasis in cancer [18,19,21,22,23]. A better understanding of the mechanisms underlying anoikis resistance and anchorage-independent growth can offer new opportunities to establish treatment strategies to control cancer. This review elaborates on the critical role of ncRNAs in the regulation of anoikis and anchorage-independent cell growth in cancer.

## 2. MiRNAs Positively Regulating Anoikis Resistance and Anchorage-Independent Growth

### 2.1. MiRNAs Regulating Apoptosis- and Cell Survival-Related Factors

#### 2.1.1. MiR-21-5p and miR-25-3p

One of the most profusely investigated miRNAs is miR-21-5p, whose expression is upregulated and correlated with metastasis status in several cancers [24,25,26]. A recent study also demonstrated that high expression of miR-21-5p is associated with poor overall survival and lymph node metastasis in patients with esophageal cancer. In that study, miR-21-5p was validated to target programmed cell death 4 (PDCD4) and phosphatase and tensin homolog (PTEN), thereby suppressing anoikis in vitro. It was also found that the overexpression or knockdown of miR-21-5p promotes or impedes liver metastases in vivo, respectively [27].

**Figure 1:**
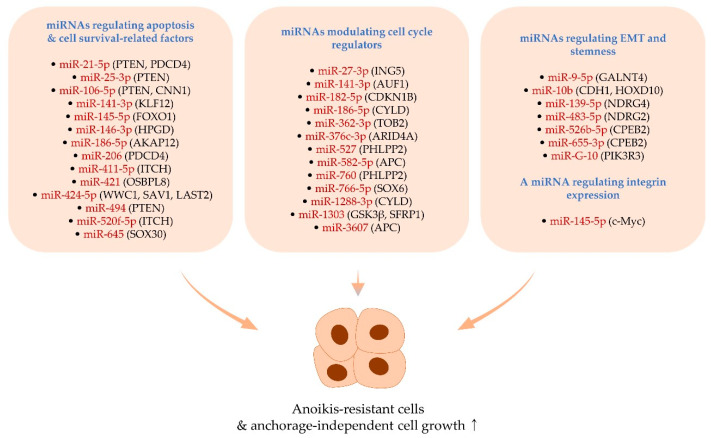
Oncogenic miRNAs (red) and their relevant target genes (black) facilitating anoikis resistance and anchorage-independent cell growth in cancer. It is described in Table 1 and Section 2.

In retinoblastoma, PTEN is also targeted by miR-25-3p (a member of the miR-106b-25 cluster), thereby activating PI3K/Akt signaling and anchorage-independent cell growth. Knockdown of miR-25-3p increases the expression of cleaved caspase-3 as well as the induction of apoptosis in vitro. Moreover, it was observed that miR-25-3p accelerates cancer growth, which can be abolished by PTEN restoration or PI3K inhibition in vivo [28] (Figure 1 and Table 1).

#### 2.1.2. MiR-106-5p and miR-141-3p

It has been noticed that miR-106-5p (a member of the miR-106b-25 cluster) is overexpressed in pituitary adenoma tissues compared to their normal counterparts and that miR-106-5p is able to augment the migration, invasion, and anchorage-independent growth of cancer cells by directly regulating the expression of PTEN [32]. There is consistent evidence that miR-106-5p can promote lung metastasis of breast cancer by targeting calponin 1 (CNN1), which enhances apoptosis rate and inactivates Rho-associated coiled-coil containing protein kinase 1 (ROCK1) in breast cancer cells [60]. However, miR-106-5p inhibits the metastasis of colorectal cancer [61], implying that miR-106-5p behaves differently depending on the type of cancer (Figure 1 and Table 1).

The relationship between miR-141-3p and metastasis has been explored in cancer. For example, exosomal miR-141-3p derived from prostate cancer cells activates osteogenesis, thus facilitating the bone metastasis of prostate cancer [62]. Also, exosomal miR-141-3p was identified as a possible biomarker for metastatic progression of rectal cancer since high levels of exosomal miR-141-3p in plasma from cancer patients display the correlation with the degree of liver metastasis [63]. In addition, miR-141-3p is overexpressed in ovarian cancer tissues, and this miRNA can augment anchorage-independent cell growth and anoikis resistance by suppressing the expression of Kruppel-like factor 12 (KLF12), which interferes with Sp1-activated transcription of the survivin gene. It was also observed that miR-141-3p stimulates the growth of metastatic ovarian cancer cells in vivo [34] (Figure 1 and Table 1).

#### 2.1.3. MiR-145-5p and miR-146-3p

Forkhead box O1 (FOXO1) is known as an anoikis-promoting cellular factor and suppresses the anchorage-independent growth of cancer cells [9]. A recent study demonstrated that miR-145-5p levels are upregulated in cancer tissues procured from bladder cancer patients with lymph node metastasis. In metastatic bladder cancer cells, miR-145-5p directly targets FOXO1, hence triggering the anchorage-independent growth of cancer cells in vitro and cancer growth in vivo [37]. Interestingly, miR-145-5p hampers the proliferation of non-metastatic cancer cells by suppressing the activation of signal transducer and activator of transcription 3 (STAT3) [37], suggesting that the status of STAT3 activation can switch the function of miR-145-5p in cancer (Figure 1 and Table 1).

15-hydroxyprostaglandin dehydrogenase (HPGD) serves as a tumor suppressor by converting prostaglandins into inactive forms and inhibiting prostaglandin-endoperoxide synthase [38,64]. In cervical cancer, HPGD suppresses cell proliferation, migration, and anchorage-independent cell growth by negatively regulating STAT3 and Akt activations [38]. In that study, it was also denoted that human papillomavirus E6/E7 oncoproteins increase the level of miR-146-3p, which targets HPGD and supports the anchorage-independent growth of cancer cells [38] (Figure 1 and Table 1).

#### 2.1.4. MiR-186-5p, miR-206, and miR-421

The analysis of miRNA profiling revealed that miR-186-5p is upregulated in serum from patients with prostate cancer and in metastatic cancer cell lines [40]. The knockdown of miR-186-5p attenuates anchorage-independent growth, along with a decrease in Akt activity. Further analyses indicate that miR-186-5p directly modulates the expression of A-kinase anchoring protein 12 (AKAP12) [40], which inhibits PI3K/Akt activity [40,65] (Figure 1 and Table 1).

Kruppel-like factor 4 (KLF4) can act as an oncogenic or a tumor-suppressive transcription factor depending on the type of cancer. For example, KLF4 represses EMT, invasion, and metastasis in lung and pancreatic cancer [66,67]. By contrast, KLF4 enhances the stemness of osteosarcoma cells by activating p38 signaling, conferring metastatic potential to cancer cells [68]. In breast cancer, KLF4 is enriched in breast cancer stem cells and transcriptionally increases miR-206 that targets PDCD4. Both KLF4 and miR-206 act as pro-survival factors and promote anoikis resistance and tumorigenesis in breast cancer [42] (Figure 1 and Table 1).

Oxysterol binding protein-like 8 (OSBPL8, also known as ORP8) triggers apoptosis induction by releasing cytochrome c from mitochondria and sensitizes cancer cells to Fas-mediated cell death [46,69]. In lung cancer, the overexpression of OSBPL8 hinders anchorage-independent growth. Further evidence indicated that miR-421 is overexpressed in lung cancer cells and targets OSBPL8, suggesting that miR-421 can act as an anoikis repressor in lung cancer [46] (Figure 1 and Table 1).

#### 2.1.5. MiR-411-5p and miR-520f-5p

Itchy E3 ubiquitin-protein ligase (ITCH, also known as AIP4) positively regulates death receptor-mediated apoptosis via promoting the degradation of CASP8 and FADD-like apoptosis regulator (CFLAR, also called c-FLIP), an anti-apoptotic factor [70]. Further, ITCH can inhibit the expression of C-X-C motif chemokine receptor 4 (CXCR4), which promotes the metastasis of cancer cells [71]. Moreover, recent studies demonstrated that miR-411-5p and miR-520f-5p levels are upregulated in hepatocellular carcinoma and melanoma tissues, respectively. These miRNAs intensify the anchorage-independent growth of cancer cells via targeting ITCH [45,49] (Figure 1 and Table 1).

#### 2.1.6. MiR-424-5p

The Hippo pathway primarily consists of mammalian STE20-like protein kinase 1 and 2 (MST1/2) and large tumor suppressor kinase 1 and 2 (LATS1/2), which partners with MOB kinase activator 1 (MOB1). MST1/2 forms a complex with Salvador family WW domain-containing protein 1 (SAV1), and this complex activates LATS1/2-MOB1 complexes, ultimately leading to the inactivation of Yes-associated protein 1 (YAP1) [72,73,74]. In addition, YAP1 is also inactivated by WW and C2 domain containing 1 (WWC1, also called KIBRA), which activates LATS1/2 [75]. Furthermore, it was demonstrated that YAP1 inhibits apoptosis by promoting the expression of anti-apoptotic genes such as BCL-2 and that the Hippo pathway can be activated by cell detachment, thus constraining YAP1 activation and consequently inducing anoikis [76]. Recent evidence further showed that miR-424-5p prompts anoikis resistance and lung metastasis of thyroid cancer by inactivating the Hippo pathway via concurrently targeting WWC1, SAV1, and LAST2 [47] (Figure 1 and Table 1).

#### 2.1.7. MiR-494 and miR-645

The expression of miR-494 is upregulated in colorectal cancer tissues, and this miRNA can promote cancer progression by negatively regulating adenomatous polyposis coli (APC) [77]. Additionally, it was found that miR-494 potentiates the anchorage-independent growth of colorectal cancer cells by targeting PTEN [48]. Further analyses indicated that miR-494 expression is subdued by nuclear factor kappa B subunit 2 (NFKB2, also named p100) that interacts with and inactivates ERK2, consequently inhibiting c-Jun, a transcription factor of miR-494 [48]. Anchorage-independent cell growth resulted from the knockdown of NFKB2 can be reversed by miR-494 inhibition [48], supporting that miR-494 undeviatingly promotes cancer progression (Figure 1 and Table 1).

Screening of miRNAs identified that miR-645 is one of the upregulated miRNAs in colorectal cancer tissues [53]. The overexpression of miR-645 attenuates apoptosis caused by anti-cancer agents such as cisplatin in colorectal cancer cells and promotes cancer growth in vivo. Also, the anchorage-independent growth of normal human epithelial colon cells is significantly reinforced by miR-645 owing to its ability to target SRY-box transcription factor 30 (SOX30) [53], a pro-apoptotic factor [78]. In line with this, another study also denoted that miR-645 facilitates liver metastasis of colorectal cancer in a mouse xenograft model [79] (Figure 1 and Table 1).

### 2.2. MiRNAs Modulating Cell Cycle Regulators

#### 2.2.1. MiR-27-3p

Several studies demonstrated that miR-27-3p promotes and suppresses the metastasis of pancreatic cancer and hepatocellular carcinoma, respectively [80,81], suggesting the dual role of this miRNA in a cellular context-dependent manner. Another study showed that the anchorage-independent growth of osteosarcoma cells is accelerated by miR-27-3p, whose expression is upregulated in cancer cells in comparison with normal osteocyte cells [31]. A further study on the mechanism of miR-27-3p indicated that G1-S cell cycle progression is promoted by miR-27-3p that targets the inhibitor of growth family member 5 (ING5). The inhibition of miR-27-3p leads to a decrease in CCND1 levels and an increase in cyclin-dependent kinase inhibitor 1A (CDKN1A, also known as p21Cip1) and CDKN1B (also known as p27Kip1). Ectopic overexpression of ING5 in miR-27-3p-overexpressing cells attenuates anchorage-independent growth, together with an increase in CDKN1A and CDKN1B levels [31] (Figure 1 and Table 1).

#### 2.2.2. MiR-141-3p

In addition to the regulation of KLF12 by miR-141-3p (Section 2.1.2), this miRNA also targets AU-rich element RNA-binding protein 1 (AUF1, also named HNRNPD) [35], which can decay CCND1 mRNAs [82]. Additional evidence showed that miR-141-3p expression is repressed by tumor protein P63 alpha containing the transactivation domain (TAp63α), leading to a reduction of CCND1. Besides, TAp63α diminishes in vitro anchorage-independent growth and in vivo tumorigenic growth of bladder cancer [35]. TAp63α and CCND1 are considered to suppress metastasis and anoikis, respectively [9,83,84]. The TAp63α/miR-141-3p/AUF1/CCND1 axis can support the finding that TAp63α inhibits cancer metastasis [83,84] and that miR-141-3p serves as an anoikis-resistant factor in cancer (also see Section 2.1.2) (Figure 1 and Table 1).

#### 2.2.3. MiR-182-5p and miR-1303

It was demonstrated that miR-182-5p acts as an oncogenic factor, inhibits apoptosis, and advances cell proliferation, migration, and invasion by activating Wnt/β-catenin signaling in bladder cancer [85]. This finding suggests the feasibility that miR-182-5p confers resistance to anoikis in bladder cancer cells via Wnt/β-catenin, which can inhibit anoikis [86]. Also, it was noted that miR-182-5p promotes anchorage-independent growth by targeting CDKN1B in bladder cancer cells [39] (Figure 1 and Table 1). Several studies showed that CDKN1B obstructs anchorage-independent cell growth in cancer [87,88,89]. In terms of anoikis, CDKN1B overexpression causes anoikis resistance in immortalized mammary epithelial cells [90]. However, it was shown that anoikis is unaffected by RNAi-mediated CDKN1B silencing in esophageal cancer cells [91]. Such discrepancy could be due to the use of different cell types and also implies the possibility that CDKN1B regulates anchorage-independent growth in cancer cells that already acquire anoikis resistance mediated by other factors.

By targeting Dickkopf WNT signaling pathway inhibitor 3 (DKK3), miR-1303 is known to activate Wnt/β-catenin signaling and facilitate prostate cancer progression by regulating proliferation, migration, and invasion [92]. Furthermore, in neuroblastoma cells, it was noted that miR-1303 targets both glycogen synthase kinase-3 beta (GSK3β) and secreted frizzled-related protein 1 (SFRP1), which are endogenous inhibitors of Wnt/β-catenin. Therefore, miR-1303 can increase the level of CCND1, a downstream target of Wnt/β-catenin, and promote anchorage-independent cell growth [57] (Figure 1 and Table 1).

#### 2.2.4. MiR-186-5p and miR-1288-3p

As stated in Section 2.1.4, miR-186-5p is involved in the regulation of anchorage-independent growth of cancer cells by modulating the AKAP12/PI3K/Akt axis. Besides, miR-186-5p was found to target CYLD lysine 63 deubiquitinase (CYLD) [41], which inhibits nuclear factor kappa B (NF-κB)-dependent CCDN1 expression [93]. The high expression of miR-186-5p was observed in melanoma tissues, and miR-186-5p overexpression triggers the anchorage-independent growth of melanoma cells [41] (Figure 1 and Table 1).

It was also denoted that miR-1288-3p is upregulated in glioblastoma tissues and cell lines compared to normal brain tissues and astrocyte cells, respectively [56]. Ectopic expression of miR-1288-3p boosts the anchorage-independent growth of glioblastoma cells by targeting CYLD [56] (Figure 1 and Table 1).

#### 2.2.5. MiR-362-3p and miR-376c-3p

Transducer of ErbB-2 2 (TOB2) was identified to suppress cell cycle progression from G1 to S phases, at least partly through restraining the transcription of CCND1 [94,95]. Additionally, a recent study found that the overexpression or knockdown of miR-362-3p increases or decreases the anchorage-independent growth of hepatocellular carcinoma cells, respectively, through targeting TOB2. Further, the expression of TOB2 is downregulated in hepatocellular carcinoma tissues, and the anchorage-independent growth of hepatocellular carcinoma cells is also hampered by TOB2 overexpression [43] (Figure 1 and Table 1).

E2F transcription factors (E2F) control multiple events, including cell cycle as well as cancer metastasis by regulating various genes, such as cell division cycle 6 (CDC6) and VEGFA [96,97]. It was illustrated that miR-376c-3p augments the anchorage-independent growth of gastric cancer cells via repressing AT-rich interactive domain-containing protein 4A (ARID4A, also called RBP-1) [44], which negatively regulates E2F-mediated transcription [98] (Figure 1 and Table 1). Additionally, miR-376c-3p was validated to promote metastasis of hepatocellular carcinoma cells in vivo [67].

#### 2.2.6. MiR-527 and miR-760

The expression of miR-527 and miR-760 is upregulated in esophageal and ovarian cancer tissues, respectively, compared to their adjacent normal tissues [51,54]. Functional experiments showed that the anchorage-independent growth ability of cancer cells is enhanced by overexpressing either miR-527 or miR-760, in company with an increase in CCND1 levels (Figure 1 and Table 1). Besides, it was identified that both miRNAs target PH domain leucine-rich repeat-containing protein phosphatase 2 (PHLPP2), a negative regulator of cell cycle progression [51,54].

#### 2.2.7. MiR-582-5p and miR-3607

Adenomatous polyposis coli (APC) participates in the regulation of cell cycle at multiple points by, for example, reducing the level of CCND1 [99,100,101]. Also, APC can downregulate the level of β-catenin, which negatively controls anoikis [102]. Recent investigations showed that APC is directly regulated by miR-582-5p and miR-3607 in colorectal and lung cancer, respectively [52,58]. The upregulation of these miRNAs can increase anchorage-independent cell growth and CCND1 levels, whereas the knockdown of these miRNAs achieves the opposite outcomes [52,58] (Figure 1 and Table 1).

#### 2.2.8. MiR-766-5p

SRY-box transcription factor 6 (SOX6) levels are inversely correlated with metastasis in cancer, and CDKN1A and CCND1 are upregulated and downregulated, respectively, by SOX6, suggesting that its tumor-suppressive function is associated with cell cycle regulation [103,104]. Recently, it was reported that the inhibition of miR-766-5p blocks cell proliferation, invasion, and survival of SW480 colorectal cancer cells [105]. Moreover, it was denoted that miR-766-5p can enhance the anchorage-independent growth of SW480 colorectal cancer cells partly via regulating SOX6 [55] (Figure 1 and Table 1). On the other hand, miR-766-5p exerts a tumor-suppressive effect by inhibiting cell proliferation in Caco-2 colorectal cancer cells [106]. Since it was proven that autophagy blockade decreases and increases the proliferation of Caco-2 and SW480 cells, respectively [107], miR-766-5p may directly or indirectly regulate the expression and activity of autophagy-related factors, thereby functioning differently depending on the cell type.

### 2.3. MiRNAs Regulating EMT and Stemness

#### 2.3.1. MiR-9-5p

It has been reported that miR-9-5p augments cell growth, angiogenesis, EMT, and metastasis in several cancers, such as cervical and lung cancer [108,109]. In hepatocellular carcinoma, miR-9-5p levels are elevated, and this miRNA enhances the stemness and anoikis resistance of cancer cells by targeting polypeptide N-acetylgalactosaminyltransferase 4 (GALNT4) (Figure 1 and Table 1). Further investigations showed that GALNT4 attenuates the stemness and anchorage-independent survival of hepatocellular carcinoma cells in vitro and inhibits cancer cell growth in vivo partly via inactivating epidermal growth factor receptor (EGFR) [29].

#### 2.3.2. MiR-10b

By targeting multiple genes, miR-10b augments EMT and metastasis in various cancer types. For instance, miR-10b targets CDH1 and triggers EMT in laryngeal carcinoma [110]. Also, miR-10b can increase the levels of pro-metastatic genes by targeting homeobox D10 (HOXD10), a metastasis suppressor [111]. In melanoma, serine/threonine-protein kinase B-Raf (BRAF) gene is frequently mutated, and BRAF^V600E^ is the most common type of BRAF [112]. The expression of miR-10b is transcriptionally activated by BRAF^V600E^-mediated upregulation of Twist family BHLH transcription factor 1 (TWIST1) in melanoma cells. Importantly, miR-10b elevates the anchorage-independent growth activity of melanoma cells [30] (Figure 1 and Table 1).

#### 2.3.3. MiR-139-5p and miR-483-5p

The levels of both miR-139-5p and miR-483-5p are upregulated in adrenocortical cancer tissues and inversely correlated with the overall survival of patients [33]. These miRNAs promote EMT, migration, invasion, and anchorage-independent cell growth in adrenocortical cancer without affecting cell cycle as well as apoptosis. Evidence provided indicated that miR-139-5p and miR-483-5p target N-Myc downstream-regulated gene 4 (NDRG4) and NDRG2, respectively, and that overexpression of either NDRG4 or NDRG2 inhibits the invasive capacity of cancer cells [33] (Figure 1 and Table 1). NDRG4 and NDRG2 have also been regarded to exhibit the tumor suppressive function by inhibiting cell proliferation, migration, invasion, and metastasis in different types of cancer, such as glioblastoma and breast cancer [113,114,115]. However, it was validated that NDRG2 can support liver metastasis of cancer by switching macrophage phenotype from M1-like tumor-associated macrophages (TAMs) to M2-like TAMs [116], suggesting that further investigations are necessary to unveil the precise function of NDRG2.

#### 2.3.4. MiR-526b-5p and miR-655-3p

Splice variant isoforms of cytoplasmic polyadenylation element-binding protein 2 (CPEB2) function differently in breast cancer. For example, CPEB2A and CPEB2B act as a tumor suppressor and an oncogene, respectively, and a high CPEB2B/CPEB2A ratio leads to anoikis resistance and metastasis of breast cancer [117,118]. In addition, the downregulation of CPEB2A stimulates EMT, stemness, anchorage-independent growth, and tumorigenesis [50]. Further, both miR-526b-5p and miR-655-3p were identified to directly target CPEB2, suggesting a possibility that these miRNAs can serve as anoikis-resistant factors (Figure 1 and Table 1), even though more investigation on the effect of these miRNAs especially on the regulation of CPEB2B/CPEB2A ratio is warranted.

#### 2.3.5. MiR-G-10

Deep sequencing analysis of GRSF1-bound miRNAs identified that miR-G-10 is one of the most abundant miRNAs in cervical cancer. A further investigation on the molecular mechanism of miR-G-10 indicated that the malignant phenotypes of cancer cells, such as EMT and anoikis resistance, are promoted by miR-G-10 that increases the expression of phosphatidylinositol 3-kinase regulatory subunit gamma (PIK3R3), an upstream activator of NF-κB. Indeed, lung metastasis of cervical cancer is accelerated by miR-G-10 in vivo [59] (Figure 1 and Table 1).

### 2.4. A miRNA Regulating Integrin Expression

#### MiR-145-5p

As a pleiotropic transcription factor, c-Myc controls diverse genes that participate in myriad cellular events, such as cell proliferation, cellular transformation, EMT, and stemness [119,120,121]. In terms of cancer metastasis, although c-Myc can trigger metastasis, the susceptibility of cancer cells to TNF-related apoptosis-inducing ligand (TRAIL) is also augmented by c-Myc overexpression [122]. Moreover, paradoxically, c-Myc has been reported to constrain cancer metastasis via the transcriptional repression of integrin subunit alpha V (ITGAV) and ITGB3 subunits [123]. Since these integrin subunits can protect cells from anoikis induction, it is feasible that c-Myc-regulating miRNAs can modulate anoikis resistance and metastasis [124]. Recent evidence showed that miR-145-5p escalates the level of ITGAV and ITGB3 subunits via targeting c-Myc in esophageal cancer cells, thereby enhancing in vitro anoikis resistance and in vivo metastasis [36] (Figure 1 and Table 1).

## 3. MiRNAs Suppressing Anoikis Resistance and Anchorage-Independent Growth

### 3.1. MiRNAs Regulating Apoptosis- and Cell Survival-Related Factors

#### 3.1.1. MiR-10a-5p and miR-22-3p

**Figure 2:**
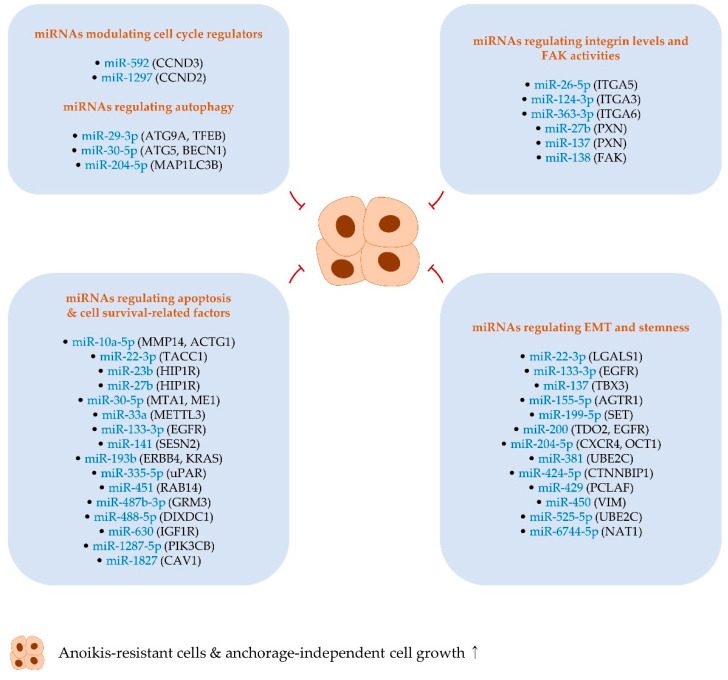
Tumor-suppressive miRNAs (blue) and their relevant target genes (black) suppressing anoikis resistance and anchorage-independent cell growth in cancer. It is described in Table 2 and Section 3.

In colorectal cancer, miR-10a-5p is able to induce anoikis in vitro and suppress metastasis in vivo. Further evidence revealed that miR-10a-5p targets matrix metallopeptidase 14 (MMP14) and actin gamma 1 (ACTG1), both of which positively regulate BCL-2 levels and render cancer cells resistant to anoikis in colorectal cancer cells [125] (Figure 2 and Table 2).

Tumor-suppressive miR-22-3p is underexpressed in rhabdomyosarcoma tissues, and the reconstitution of miR-22-3p was observed to induce apoptosis and subdue anchorage-independent growth of cancer cells. Further, it was disclosed that miR-22-3p exerts its tumor-suppressive function through targeting transforming acidic coiled-coil containing protein 1 (TACC1), which reinforces anchorage-independent growth and protects cells from apoptosis via elevating the level of PI3K [126,127] (Figure 2 and Table 2).

**Table 2:** Tumor-suppressive miRNAs that suppress the anoikis resistance and anchorage-independent growth of cancer.

miRNA	Type of Cancer	In Vitro Experiment	In Vivo Experiment	Ref.
miR-10a-5p	Colorectal cancer	Plating cells on polyHEMA-coated plates + annexin-V/propidium iodide analysis *	Injection of pri-miR-10a-expressing SW620 cells into the spleen	[125]
miR-22-3p	Hepatocellular carcinoma	Soft agar assay **	Orthotopic implantation of galectin-1 silencing MHCC97L cells into the liver	[128]
miR-22-3p	Rhabdomyosarcoma	Soft agar assay **	Subcutaneous injection of RD18 cells conditionally expressing miR-22-3p	[127]
miR-23b	Prostate cancer	Soft agar assay **	Orthotopic injection of PC3-ML cells overexpressing miR-23b and miR-27b	[129]
miR-26-5p	Hepatocellular carcinoma	Plating cells on polyHEMA-coated plates + annexin-V/propidium iodide analysis *	Intravenous injection of BEL-7404 cells overexpressing miR-26 for in vivo anoikis assay	[130]
miR-27b	Colorectal cancer	Colony formation assay **	Subcutaneous injection of HCT116 cells overexpressing miR-27-3p	[131]
miR-27b	Prostate cancer	Soft agar assay **	Orthotopic injection of PC3-ML cells overexpressing miR-23b and miR-27b	[129]
miR-29-3p	Pancreatic cancer	Soft agar assay **	-	[132]
miR-30-5p	Hepatocellular carcinoma	Plating cells on polyHEMA-coated plates + ethidium homodimer-1 assay *	Examination of malignant ascites for the measure of anoikis. Tail vein injection of HCCLM3 cells overexpressing miR-30-5p for lung metastasis assays	[133]
miR-30-5p	Colorectal cancer	Soft agar colony assay **	-	[134]
miR-30-5p	Renal cell carcinoma	Soft agar assay **	Subcutaneous injection of Caki-1 cells transfected with miR-30-5p	[135]
miR-33a	Lung cancer	Soft agar colony formation assay **	-	[136]
miR-124-3p	Colorectal cancer	Plating cells on polyHEMA-coated plates + cell viability assay using 0.4% trypan blue *	Tail vein injection of miR-124-3p-overexpressing Lovo and SW620 cells	[137]
miR-133-3p	Esophageal cancer	Plating cells on polyHEMA-coated plates + annexin-V/propidium iodide analysis *	Tail vein injection of KYSE150 and ECa109 cells expressing miR-133-3p	[138]
miR-133-3p	Prostate cancer	Spheroid formation assay using ultra-low cluster plates *	Inoculation of PC-3 cells into the left cardiac ventricle + tail vein injection of miR-133-3p inhibitors	[139]
miR-137	Melanoma	Soft agar assay **	-	[140]
miR-137	Pancreatic cancer	Plating cells on polyHEMA-coated plates + annexin-V/propidium iodide analysis *	Intraperitoneal injection of PANC-1 cells overexpressing miR-137 for in vivo anoikis assay	[141]
miR-138	Ewing’s sarcoma	Annexin-V/propidium iodide analysis following miR-138 transfection *	Tail vein injection of SKES1 cells transfected with miR-138	[142]
miR-141	Endometrial cancer	Culturing cells on anchorage-resistant plates + calcein AM assay *	-	[143]
miR-155-5p	Glioblastoma	Soft agar colony formation assay **	Injection of miR-155-expressing SNB19 cells into dorsal flank	[144]
miR-193b	Pancreatic cancer	Colony-formation assay **	-	[145]
miR-193b	Ewing’s sarcoma	Soft agar assay **	-	[146]
miR-199-5p	Colorectal cancer	Colony-forming assay **	-	[147]
miR-200	Breast cancer	Soft agar assay **	-	[148]
miR-200	Bladder cancer	Soft agar assay **	-	[149]
miR-204-5p	Gastric cancer	Plating cells on polyHEMA-coated plates + annexin-V/propidium iodide analysis *	-	[150]
miR-204-5p	Medulloblastoma	Soft agar colony formation assay **	-	[151]
miR-335-5p	Gastric cancer	Soft agar colony formation assay **	-	[152]
miR-363-3p	Papillary thyroid cancer	Plating cells on polyHEMA-coated plates + apoptosis analysis by flow cytometry *	-	[153]
miR-381	Rectal cancer	Soft agar assay **	-	[154]
miR-424-5p	Hepatocellular carcinoma	Plating cells on polyHEMA-coated plates + Western blotting for measuring caspase 3 *	Intra-tumoral injection of miR-424-5p expression vectors in a subcutaneous SMMC7721 xenograft model	[155]
miR-429	Soft tissue sarcoma	Soft agar colony formation assay **	-	[156]
miR-450	Ovarian cancer	Plating cells in ultra-low attachment plates + annexin-V assay *	Intraperitoneal injection of A2780 cells expressing miR-450	[157]
miR-451	Osteosarcoma	Plating cells on polyHEMA-coated plates + annexin-V/propidium iodide analysis *	Tail vein injection of osteosarcoma cells overexpressing miR-451	[158]
miR-487b-3p	Colorectal cancer	Soft agar assay **	Subcutaneous injection of CBS cells expressing miR-487b precursor	[159]
miR-488-5p	Melanoma	Soft agar assay **	-	[160]
miR-525-5p	Cervical cancer	Plating cells on polyHEMA-coated plates + annexin-V/propidium iodide analysis *	-	[161]
miR-592	Colorectal cancer	Soft agar assay **	-	[162]
miR-630	Breast cancer	Plating cells on polyHEMA-coated plates + Alamar blue dye assay *	-	[163]
miR-1287-5p	Breast cancer	Sphere assay using ultra-low attachment plates **	Subcutaneous injection of SUM159 cells overexpressing miR-1287-5p	[164]
miR-1297	Colorectal cancer	Colony formation assay **	-	[165]
miR-1827	Lung cancer	Plating cells on polyHEMA-coated plates + annexin-V/propidium iodide analysis *	-	[166]
miR-6744-5p	Breast cancer	Plating cells on low adhesion plates + caspase-3/7 activity assay *	Microinjection of miR-6744-5p transfected MDA-MB-231 cells into the yolk of zebrafish embryos	[167]

* anoikis assays. ** assays for the measurement of anchorage-independent growth.

#### 3.1.2. MiR-23b and miR-27b

Huntingtin-interacting protein 1-related (HIP1R) is involved in the encouragement of cell survival via stabilizing receptor tyrosine kinases; therefore, loss-of-function mutants of HIP1R are able to induce cell death [168]. A recent study has shown that both miR-23b and miR-27b, members of the miR-23b-27b-24-1 cluster, target HIP1R, inhibit anchorage-independent growth, and suppress in vivo prostate cancer metastasis [129] (Figure 2 and Table 2). However, HIP1R can activate the anticancer immunity of T cells via lysosomal degradation of programmed cell death 1 ligand 1 (PD-L1) [169], implying that miR-23b and miR-27b may have a possibility to compromise cancer immune surveillance in vivo.

#### 3.1.3. MiR-30-5p and miR-33a

Recent studies showed that the expression of BCL-2 and BCL2 like 1 (BCL2L1, also called BCL-XL) can be positively modulated by methyltransferase-like 3 (METTL3) and metastasis-associated 1 (MTA1), respectively [170,171]. Moreover, both MTA1 and METTL3 are realized to accelerate cancer metastasis [172,173,174]. Additional studies provided evidence that miR-30-5p and miR-33a target MTA1 and METTL3, respectively, hence contributing to attenuated anchorage-independent growth of cancer cells [135,136] (Figure 2 and Table 2).

Malic acid 1 (ME1) is responsible for nicotinamide adenine dinucleotide phosphate (NADPH) production. Knockdown of ME1 increases the level of reactive oxygen species (ROS), leading to the inhibition of cell growth and the induction of anoikis [175,176]. In the case of miR-30-5p, this miRNA also targets ME1, promotes apoptosis, and attenuates the anchorage-independent growth of colorectal cancer cells [134] (Figure 2 and Table 2).

#### 3.1.4. MiR-133-3p and miR-141

The expression of miR-133-3p is downregulated in prostate cancer and further reduced in metastatic prostate cancer, suggesting that miR-133-3p may affect cellular events/signaling associated with metastasis [139]. Investigations of miR-133-3p in relation to anoikis and metastasis showed that this miRNA diminishes the level of anti-apoptotic factors (e.g., BCL-2) and the activity of PI3K/Akt signaling via targeting multiple genes (e.g., EGFR), eventually alleviating anoikis resistance and bone metastasis of prostate cancer [139] (Figure 2 and Table 2).

It was pointed out that sestrin 2 (SESN2) is upregulated and protects cancer cells from oxidative stress-induced anoikis upon detachment of cells from ECM. The silencing of SESN2 leads to anoikis in vitro and restricts distant metastasis in vivo [177]. In endometrial cancer, SESN2 was validated to be targeted by miR-141, a member of the miR-200 family. The overexpression of miR-141 induces anoikis [143], suggesting that the anoikis-inducing activity of miR-141 is partly mediated by the modulation of SESN2 and oxidative stress (Figure 2 and Table 2).

#### 3.1.5. MiR-193b

One of the characteristics of Ewing’s sarcoma is the chromosome translocation that joins Ewing’s sarcoma breakpoint region 1 (EWS) to Friend leukemia virus integration 1 (FLI1). EWS–FLI1 has been known to support anchorage-independent growth and metastasis in Ewing’s sarcoma [178,179]. Besides, screening of the effect of miRNAs repressed by EWS–FLI1 on anchorage-independent cell growth identified that miR-193b suppresses anchorage-independent growth via targeting Erb-B2 receptor tyrosine kinase 4 (ERBB4). This finding suggests that anchorage-independent growth of Ewing’s sarcoma is modulated via the EWS–FLI1/miR-193b/ERBB4 axis [146].

Kirsten rat sarcoma 2 viral oncogene homolog (KRAS) plays an important role in the metastasis of pancreatic cancer via affecting multiple cellular events such as apoptosis [180,181]. The level of miR-193b, which targets KRAS, is downregulated in pancreatic cancer. The overexpression of miR-193b activates caspase-3, thus weakening anchorage-independent growth [145] (Figure 2 and Table 2).

#### 3.1.6. MiR-335-5p and miR-451

Accumulating evidence reveals that urokinase-type plasminogen activator (uPA) and its receptor (uPAR) aggravate invasion and metastasis, for example, by degrading the element of ECM. Besides, uPAR can trigger cell migration, EMT process, and cancer stemness by activating cellular signaling factors such as ERK [182,183,184,185]. Also, it was found that the transcriptional activation of BCL2L1 via uPA/uPAR-dependent activations of ERK and PI3K/Akt contributes to anoikis resistance [186]. Moreover, it was uncovered that miR-335-5p, which is downregulated in gastric cancer, counteracts the stemness and anchorage-independent growth of gastric cancer cells through targeting uPAR [152] (Figure 2 and Table 2).

The mechanism by which miR-451 regulates anoikis resistance was investigated in osteosarcoma. Ectopic expression of miR-451 facilitates anoikis induction, and the overexpression of Ras-related protein Rab-14 (RAB14), a target of miR-451, substantially abrogates the effect of miR-451 on anoikis. These results indicate that the anoikis-promoting activity of miR-451 is partly through repressing RAB14 in osteosarcoma cells [158] (Figure 2 and Table 2). Consistent with this finding, RAB14 has been discerned to activate Akt signaling and confer metastatic potentiality in cancer cells [187,188].

#### 3.1.7. MiR-487b-3p and miR-488-5p

Liver metastasis of colorectal cancer is restrained by miR-487b-3p that inhibits the KRAS/Akt signaling pathway [189]. In another study, miR-487b-3p was also demonstrated to dampen colorectal cancer tumorigenesis by diminishing anchorage-independent growth and Akt activity. In this study, glutamate metabotropic receptor 3 (GRM3) was confirmed as a target gene of miR-487b-3p [159].

Moreover, it was noted that miR-488-5p is downregulated in melanoma and that the ectopic introduction of miR-488-5p was determined to induce apoptosis and inhibit anchorage-independent cell growth via targeting DIX domain-containing 1 (DIXDC1) [160], a pro-survival and -metastatic factor [190,191,192] (Figure 2 and Table 2).

#### 3.1.8. MiR-630 and miR-1287-5p

Anchorage-independent survival of cancer cells is reinforced in docetaxel-resistant cells, denoting that drug resistance is coupled with the induction of anoikis resistance [193]. Another study also showed that miR-630 levels are dropped in lapatinib-resistant breast cancer cells. In drug-sensitive parental cells, the inhibition of miR-630 attenuates anoikis, and the overexpression of miR-630 induces anoikis in resistant cells by targeting insulin-like growth factor 1 receptor (IGF1R) [163] (Figure 2 and Table 2).

It was validated that miR-1287-5p is one of the downregulated miRNAs in breast cancer tissues, and low expression of this miRNA is correlated with poor patient prognosis [164]. In that study, it was further observed that miR-1287-5p subdues in vitro anchorage-independent cell growth and in vivo cancer growth by targeting PIK3 catalytic subunit beta (PIK3CB) [164] (Figure 2 and Table 2).

#### 3.1.9. MiR-1827

It was shown that caveolin 1 (CAV1) is strongly expressed in lung cancer and positively correlated with lung metastasis. In vitro assay from this study indicated that CAV1 promotes migration, invasion, and the expression of EMT-related factors such as snail family transcriptional repressor 1 (SNAI1) [194]. Also, CAV1 positively regulates the expression and activity of multiple oncogenic factors, including Akt, CCND1, as well as BCL-2, thus protecting cells from apoptotic cell death in lung cancer [195]. Further, CAV1 can protect lung cancer cells from anoikis by preventing ubiquitin-mediated degradation of myeloid cell leukemia sequence 1 (MCL1), a member of the anti-apoptotic BCL-2 family [196]. In recent times, it was proven that the ectopic expression of miR-1827 contributes to the induction of anoikis by targeting CAV1 in lung cancer [166] (Figure 2 and Table 2).

### 3.2. MiRNAs Modulating Cell Cycle Regulators

#### MiR-592 and miR-1297

The levels of both miR-592 and miR-1297 are reduced in colorectal cancer tissues. Functional experiments demonstrated that both miRNAs decelerate the anchorage-independent growth of colorectal cancer cells and that CCND3 and CCND2 are targeted by miR-592 and miR-1297, respectively [162,165] (Figure 2 and Table 2).

### 3.3. MiRNAs Regulating EMT and Stemness

#### 3.3.1. MiR-22-3p and miR-424-5p

Lectin galactoside-binding soluble 1 (LGALS1, also named galectin-1) has been recognized to support EMT and metastasis in hepatocellular carcinoma [197,198]. Lately, it was also demonstrated that the knockdown of LGALS1 reduces anchorage-independent growth and lung metastasis of hepatocellular carcinoma cells [128]. In silico analysis of miRNAs showed that miR-22-3p directly targets LGALS1, hence obstructing anchorage-independent cell growth [128] (Figure 2 and Table 2).

Analysis of miRNA profile unveiled that miR-424-5p is one of the downregulated miRNAs in anoikis-resistant hepatocellular carcinoma cells, which display elevated EMT-related factors such as CDH2 (also known as N-cadherin) [155]. Further, it was observed that overexpression of miR-424-5p causes a reversal of EMT status, induces anoikis, and suppresses the tumorigenicity of hepatocellular carcinoma by targeting beta-catenin-interacting protein 1 (CTNNBIP1, also known as ICAT), an endogenous β-catenin repressor [155] (Figure 2 and Table 2).

#### 3.3.2. MiR-133-3p, miR-137, and miR-155-5p

As stated in Section 3.1.4, miR-133-3p serves as an anoikis-promoting factor by targeting, for example, EGFR. Since EGFR can regulate cellular signaling involved in EMT [199], it is feasible that miR-133-3p modulates EMT in cancer. Indeed, it was reported that miR-133-3p suppresses EMT, eventually restricting anoikis resistance, anchorage-independent growth, and lung metastasis of esophageal cancer cells [138] (Figure 2 and Table 2).

T-box transcription factor 3 (TBX3) can provoke EMT by positively regulating SNAI1 and SNAI2 in cancer [200,201]. It was also suggested that TBX3 participates in the regulation of metastatic processes via transcriptionally downregulating CDH1 [202]. Another study showed that TBX3 is targeted by miR-137; therefore, miR-137 can hinder the migration and anchorage-independent growth of melanoma cells [140] (Figure 2 and Table 2).

Angiotensin II receptor type 1 (AGTR1), a receptor for angiotensin II, is capable of prompting EMT, anchorage-independent growth, and metastasis in cancer [203,204,205]. A further study on the miRNA-AGTR1 relationship and the molecular mechanism of AGTR1-mediated signaling showed that miR-155-5p depresses EMT process and anchorage-independent growth by attenuating the activity of NF-κB via targeting AGTR1 in glioblastoma [144] (Figure 2 and Table 2).

#### 3.3.3. MiR-199-5p and miR-200

SET nuclear proto-oncogene (SET) is positively correlated with the poor prognosis of patients with cancer and is known to promote EMT [147,206,207,208]. The silencing of SET reduces the level of EMT markers such as vimentin (VIM) and represses the migration and invasion of cancer cells [208]. In colorectal cancer, it was indicated that anchorage-independent cell growth is enhanced by the overexpression of SET. Moreover, SET is targeted by miR-199-5p, which is downregulated in colorectal cancer tissues [147]. These results suggest that low miR-199-5p expression is one of the causes of high SET levels in colorectal cancer and that miR-199-5p can negatively modulate anchorage-independent growth via targeting SET (Figure 2 and Table 2).

Tryptophan 2,3-dioxygenase (TDO2) catabolizes tryptophan, mediating the production of kynurenine, a ligand of aryl hydrocarbon receptor (AHR). The activation of AHR via the TDO2/kynurenine axis is perceived to reinforce EMT transition, anoikis resistance, and metastasis [209,210]. A recent study further demonstrated that TDO2 is targeted by miR-200 in breast cancer and that the overexpression of TDO2 brings about enhanced anchorage-independent growth [148], suggesting that miR-200 can inhibit anchorage-independent cell growth through repressing EMT process mediated by the TDO2/kynurenine/AHR signaling (Figure 2 and Table 2).

X-linked inhibitor of apoptosis (XIAP) is responsible for anoikis resistance by inhibiting apoptotic cell death in cancer [211]. Other than inhibiting apoptosis, XIAP can activate EMT process [212]. In bladder cancer, it was noticed that the expression of miR-200 is transcriptionally repressed by XIAP, which in turn upregulates the level of EGFR, a target of miR-200 [149]. In addition, the knockdown of XIAP attenuates anchorage-independent cell growth [149], suggesting that the anchorage-independent growth of bladder cancer can be modulated by the XIAP/miR-200/EGFR/EMT axis (see Section 3.3.2 for EGFR/EMT relationship) (Figure 2 and Table 2).

#### 3.3.4. MiR-204-5p

Accumulating evidence reveals that miR-204-5p hinders EMT, stemness, invasion, and metastasis in multiple types of cancer [213,214,215,216]. In gastric cancer, low levels of miR-204-5p are correlated with lymph node metastasis, and this miRNA negatively regulates C-X-C motif chemokine receptor 4 (CXCR4) [216], which can provoke metastasis by blocking anoikis [217]. Also, miR-204-5p interrupts EMT process by targeting POU class 2 homeobox 1 (POU2F1, also called OCT1) [218], which is proposed to protect cells from anoikis [219]. Further, there is consistent evidence that miR-204-5p negatively regulates EMT process and dampens anoikis resistance in gastric cancer [150]. In this study, sirtuin 1 (SIRT1) was validated as a target of miR-204-5p. However, SIRT1 functions as a tumor-suppressor by impeding migration, invasion, and stemness in gastric cancer [220,221]. Therefore, it is feasible that miR-204-5p acts as an anoikis-promoting miRNA by regulating CXCR4, OCT1, etcetera, rather than SIRT1, in gastric cancer (Figure 2 and Table 2).

#### 3.3.5. MiR-381 and miR-525-5p

Ubiquitin-conjugating enzyme E2 C (UBE2C) has been illustrated to facilitate proliferation, survival, invasion, and EMT in cancer [187,222,223]. For example, UBE2C can stimulate EMT process by activating Wnt/β-catenin signaling, PI3K/Akt signaling, and the level of Zinc finger E-Box-binding homeobox 1 (ZEB1) and ZEB2 [187,223]. In addition, it was found that UBE2C knockdown leads to the reduction of anchorage-independent growth of rectal carcinoma cells and that UBE2C is targeted by miR-381, suggesting that miR-381 can modulate anchorage-independent growth partly via UBE2C [154]. Furthermore, miR-525-5p was also identified to target UBE2C, hence repressing migration, invasion, anchorage-independent growth, and anoikis resistance in cervical cancer cells [161] (Figure 2 and Table 2).

#### 3.3.6. MiR-429 and miR-450

PCNA-clamp-associated factor (PCLAF, also known as KIAA0101 and PAF) was identified to support cell proliferation and metastasis [224]. In addition, PCLAF is known to promote EMT, stemness, and anchorage-independent cell growth via activating Wnt signaling [225]. Further investigation into the relationship between PCLAF and miRNAs indicated that miR-429 directly targets PCLAF, consequently hampering the migration, invasion, and anchorage-independent growth of soft tissue sarcoma cells [156] (Figure 2 and Table 2).

One of the most markedly downregulated miRNAs is miR-450 in ovarian cancer. Investigations on the role of miR-450 indicated that EMT-related genes are extensively changed following the overexpression of this miRNA and that VIM is directly targeted by miR-450, demonstrating its tumor-suppressive role in ovarian cancer. Further tests showed that the restoration of miR-450 forcefully increases the rate of anoikis in vitro and cancer growth in vivo [157] (Figure 2 and Table 2).

#### 3.3.7. MiR-6744-5p

N-acetyltransferase 1 (NAT1) regulates multiple cellular processes. For example, NAT1 can positively affect cell proliferation and survival by modulating the level of reactive oxygen species (ROS) [226]. Also, NAT1 reinforces EMT and metastasis by regulating CDH2 and β-catenin levels in breast cancer [227]. Moreover, the genetic or pharmacological inhibition of NAT1 retards anchorage-independent cell growth in breast cancer [228]. In recent times, miR-6744-5p was found to be downregulated in anoikis-resistant breast cancer cells, implying a possibility that this miRNA acts as an anoikis-regulating miRNA [167]. In fact, the overexpression of miR-6744-5p increases the sensitivity of breast cancer cells to anoikis, along with the abatement of invasion and metastasis capabilities, via targeting NAT1 [167] (Figure 2 and Table 2).

### 3.4. MiRNAs Regulating Autophagy

#### 3.4.1. MiR-29-3p

In pancreatic cancer, miR-29-3p sensitizes cancer cells to gemcitabine and diminishes the ability of cancer cells to grow under anchorage-independent conditions [132]. The mechanism underlying these tumor-suppressive activities of miR-29-3p involves the downregulation of autophagy-related 9A (ATG9A) and transcription factor EB (TFEB), which control the trafficking of autophagosome and lysosomal function. Therefore, the overexpression of miR-29-3p causes the impairment of autophagic flux resulting from the blockage of autophagosome-lysosome fusion [132] (Figure 2 and Table 2).

#### 3.4.2. MiR-30-5p

Several studies have highlighted the role of miR-30-5p in hepatocellular carcinoma. For instance, through targeting SNAI1, miR-30-5p impedes EMT [132]. In addition, miR-30-5p induces apoptotic cell death and retards proliferation, as well as invasion [229,230]. Moreover, it has been recently demystified that miR-30-5p abates anoikis resistance and lung metastasis in hepatocellular carcinoma cells through targeting autophagy-inducing factors, namely, ATG5 and BECN1 [133] (Figure 2 and Table 2). These findings suggest the therapeutic benefit of miR-30-5p overexpression for hepatocellular carcinoma.

#### 3.4.3. MiR-204-5p

The expression of miR-204-5p is epigenetically silenced in medulloblastoma, and low levels of this miRNA are correlated with the dismal prognosis of patients [151]. The evaluation of miR-204-5p activity denoted that miR-204-5p reduces the number of cells growing anchorage-independently, at least in part owing to its capability to inhibit autophagy via targeting microtubule-associated proteins 1A/1B light chain 3B (MAP1LC3B, also called LC3B) [151] (Figure 2 and Table 2).

### 3.5. MiRNAs Regulating Integrin Levels and FAK Activities

#### 3.5.1. MiR-26-5p, miR-124-3p, and miR-363-3p

In hepatocellular carcinoma, miR-26-5p has been perceived to suppress metastasis by regulating several cellular events, such as apoptosis and EMT [231,232,233]. Moreover, miR-26-5p stimulates the anoikis of hepatocellular carcinoma cells by directly suppressing ITGA5 [130]. These observations demonstrate that miR-26-5p is a bona fide metastasis-suppressing miRNA in hepatocellular carcinoma.

Similarly, miR-124-3p, which targets ITGA3, increases the anoikis susceptibility and inhibits the lung metastasis of colorectal cancer cells. In that study, it was also shown that ITGA3 levels are positively correlated with poor prognosis of patients with colorectal cancer and that knockdown of ITGA3 augments anoikis induction and reduces metastasis [137].

Moreover, anoikis resistance is attenuated by the overexpression of miR-363-3p, which is downregulated in papillary thyroid carcinoma. In their study, ITGA6 was validated as a target of miR-363-3p [153], suggesting that metastatic cascade of papillary thyroid carcinoma is partly regulated by the miR-363-3p/ITGA6 axis (Figure 2 and Table 2).

#### 3.5.2. MiR-27b, miR-137, and miR-138

One of the adaptor molecules connecting integrins to FAK is paxillin (PXN), which is involved in ERK activation and required for FAK-mediated anoikis suppression [234,235]. Recent studies indicated that both miR-27b and miR-137 directly target PXN. Overexpression of miR-27b negatively controls the anchorage-independent growth and in vivo growth of colorectal cancer cells [131]. In addition, miR-137 overexpression suppresses anoikis resistance in vitro and in vivo concomitant with caspase-3 activation in pancreatic cancer [141]. Moreover, FAK can be directly regulated by miR-138 in Ewing’s sarcoma. Therefore, the overexpression of miR-138 results in anoikis induction in vitro and alleviates the lung metastasis of Ewing’s sarcoma cell in vivo [142] (Figure 2 and Table 2).

## 4. Long Noncoding RNAs, Anoikis, and Anchorage-Independent Growth

### 4.1. LncRNA-ANRIL

**Figure 3:**
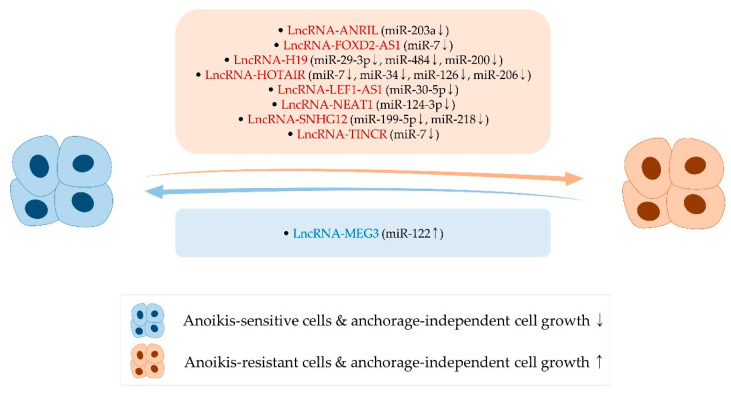
Oncogenic (red), tumor-suppressive (blue) lncRNAs, and miRNAs (black) that are affected by lncRNAs affecting anoikis and anchorage-independent cell growth in cancer. It is described in Table 3 and Section 4.

LncRNA-antisense non-coding RNA in the INK4A locus (lncRNA-ANRIL) is upregulated in several types of cancer and facilitates proliferation, survival, stemness, and metastasis [236,237,238,239,240,241]. For example, the downregulation of lncRNA-ANRIL derepresses miR-99a levels, leading to the suppression of cell survival, migration, and invasion of retinoblastoma [241]. Regarding anoikis, it was presented that the silencing of lncRNA-ANRIL leads to the activation of caspases and the induction of anoikis in glioma cells [242]. LncRNA-ANRIL knockdown increases miR-203a levels and abates the expression of several cellular factors, such as BCL-2, CDK2, c-Myc, and Akt. The knockdown of miR-203a partially reverses the effect of lncRNA-ANRIL silencing, certainly indicating that the ability of lncRNA-ANRIL to regulate anoikis is mediated by miR-203a [242] (Figure 3 and Table 3).

### 4.2. LncRNA-FOXD2-AS1

Telomerase reverse transcriptase (TERT) not only maintains telomere, but also potentiates cancer stemness and metastasis via modulating the activity of related factors, such as NF-κB and Wnt/β-catenin [252]. Also, the capacity of cancer cells to grow under anchorage-independent conditions is enhanced by TERT [253]. Further, it was recently stated that lncRNA-FOXD2 adjacent opposite strand RNA 1 (lncRNA-FOXD2-AS1) strengthens the stemness, cell survival, and anchorage-independent growth of thyroid cancer cells. LncRNA-FOXD2-AS1 sponges miR-7 that targets TERT, suggesting that anchorage-independent cell growth can be regulated by the lncRNA-FOXD2-AS1/miR-7/TERT axis [243] (Figure 3 and Table 3).

### 4.3. LncRNA-H19

It was also addressed that lncRNA-H19 imprinted maternally expressed transcript (lncRNA-H19) reinforces EMT and metastasis in diverse types of cancer. In particular, liver and lung metastases of pancreatic cancer cells are curtailed by the downregulation of lncRNA-H19 [254]. In a similar vein, lncRNA-H19 can stimulate EMT process by inhibiting miR-29-3p and miR-484, which negatively regulate STAT3 and ROCK2, respectively [255,256]. In gastric cancer, it was identified that lncRNA-H19 promotes EMT and anchorage-independent growth via suppressing the multiple tumor-suppressive miRNAs, including miR-200 [244] (also see Section 3.3.3 for miR-200) (Figure 3 and Table 3).

### 4.4. LncRNA-HOTAIR

LncRNA-HOX antisense intergenic RNA (lncRNA-HOTAIR) is strongly expressed in various cancers and can support biological processes, such as cell survival and EMT. For instance, the expression of EMT-related factors such as VIM is attenuated following the lncRNA-HOTAIR silencing. The activity of pro-survival factors, including Akt, ERK, and BCL-2, is also diminished by the downregulation of lncRNA-HOTAIR [257,258]. The oncogenic function of lncRNA-HOTAIR can be exerted by direct or indirect suppression of several tumor-suppressive miRNAs, such as miR-7, miR-34, miR-126, and miR-206 [259,260]. Consistent with these findings, it was demonstrated that lncRNA-HOTAIR promotes the anoikis resistance, anchorage-independent growth, and peritoneal/liver metastasis of gastric cancer cells [245,246] (Figure 3 and Table 3).

### 4.5. LncRNA-LEF1-AS1

LncRNA-lymphoid enhancer-binding factor 1 antisense RNA 1 (lncRNA-LEF1-AS1) is highly expressed in colorectal cancer and positively correlated with the dismal prognosis of patients [247]. In this study, it was further observed that lncRNA-LEF1-AS1 heightens the capability of colorectal cells to grow under anchorage-independent conditions and metastasize to lungs through repressing the activity of miR-30-5p to target the sex-determining region Y box 9 (SOX9) [247], which is a pro-survival, -stemness, and -metastasis factor [261,262,263]. In line with this, SOX9 was described to support anoikis resistance by promoting the stemness property of cancer cells [264] (Figure 3 and Table 3).

### 4.6. LncRNA-MEG3

LncRNA-maternally expressed 3 (lncRNA-MEG3) serves as a tumor suppressor in several cancer types, such as cervical cancer, gastric cancer, and hepatocellular carcinoma. In cervical cancer, through the ubiquitin-mediated degradation of STAT3, lncRNA-MEG3 can suppress cell survival and cancer growth [265]. Also, it was demonstrated that lncRNA-MEG3 impedes the metastasis of gastric cancer, at least partly via sponging miR-21 [266].

In hepatocellular carcinoma, lncRNA-MEG3 was found to inactivate β-catenin via transcriptionally and post-transcriptionally stimulating the expression of miR-122, thus inhibiting cancer growth in vitro and in vivo [267]. Moreover, it was denoted that lncRNA-MEG3 is epigenetically silenced in hepatocellular carcinoma. Ectopic expression of lncRNA-MEG3 brings about the retarded growth of cancer cells in the absence of anchorage [248]. The expression of lncRNA-MEG3 can be derepressed by miR-29-3p that negatively regulates the levels of DNA Methyltransferase 1 (DNMT1) and DNMT3B [248]. These findings suggest that miR-29-3p can modulate anchorage-independent growth by affecting lncRNA levels and autophagy processes (see Section 3.4.1) (Figure 3 and Table 3).

By contrast, lncRNA-MEG3 expression is upregulated following the treatment of lung cancer cells with transforming growth factor-beta (TGF-β). Consequently, the expression of miR-200 and CDH1 can be epigenetically repressed by lncRNA-MEG3 since this lncRNA is able to interact with Jumonji and AT-rich interaction domain-containing 2 (JARID2), thereby recruiting enhancer of zeste homolog 2 (EZH2) to the regulatory region of these genes. Further, it was shown that the knockdown of lncRNA-MEG3 abrogates TGF-β-induced EMT marker levels [268].

### 4.7. LncRNA-NEAT1

According to The Cancer Genome Atlas (TCGA) database, lncRNA-nuclear paraspeckle assembly transcript 1 (lncRNA-NEAT1) is upregulated or downregulated depending on the cancer types [269], implying that its role in cancer is cellular context-dependent. For instance, the apoptotic cell death of glioma cells can be promoted by lncRNA-NEAT1 that upregulates DKK3 by sponging miR-92b [270]. By contrast, the metastatic potential of endometrial cancer is heightened by lncRNA-NEAT1, which confines the activity of miR-361, an anti-metastatic miRNA [271]. In cervical cancer, lncRNA-NEAT1 levels are closely correlated with lymph node metastasis [249]. Enforced expression of lncRNA-NEAT1 diminishes miR-124-3p levels and potentiates migration, invasion, and anchorage-independent growth, along with the stimulation of EMT and NF-κB signaling (Figure 3 and Table 3). These effects of lncRNA-NEAT1 are reversed by replenishing miR-124-3p levels [249], an anoikis-promoting miRNA (see Section 3.5.1 about miR-124-3p).

### 4.8. LncRNA-SNHG12

The EMT and metastasis can be prompted by lncRNA-small nucleolar host gene 12 (lncRNA-SNHG12). In lung cancer, miR-218 is actuated following the lncRNA-SNHG12 knockdown, causing the suppression of migration, invasion, and the level of EMT-related factors, such as SNAI2 and ZEB2 [272]. Also, the knockdown of lncRNA-SNHG12 inactivates Wnt/β-catenin signaling and decreases CCND1 expression, eventually limiting the metastasis of thyroid cancer in vivo [273]. Likewise, overexpression of lncRNA-SNHG12 weakens the miR-199-5p-mediated suppression of hypoxia-inducible factor 1 alpha (HIF1α), supporting anchorage-independent growth, cell survival, and invasion in vitro and the in vivo growth of renal cancer [250] (Figure 3 and Table 3).

### 4.9. LncRNA-TINCR

In breast cancer, miR-7 performs multiple tumor-suppressive functions. Various events, such as EMT, invasion, angiogenesis, and metastasis, are adversely regulated by miR-7 owing to its ability to target oncogenes, including KLF4 and the p65 subunit of NF-κB (known as RELA proto-oncogene, NF-κB subunit (RELA)) [274,275,276]. These activities of miR-7 can be dampened by terminal differentiation-induced lncRNA (lncRNA-TINCR), whose expression is incremented by Sp1 transcription factor in breast cancer. The silencing of lncRNA-TINCR suppresses the cell survival, migration, invasion, anchorage-independent growth, and in vivo growth of breast cancer cells [251] (Figure 3 and Table 3).

## 5. Conclusions

A series of events, including anoikis resistance, are involved in the pathogenesis of metastasis. In light of this, targeting salient molecular determinants and signaling pathways can effectively manage cancer. The evidence delineated here indicates that the inhibition or restoration of ncRNAs (ncRNA-based therapy), is an attractive strategy for overcoming anoikis resistance, anchorage-independent cell growth, and metastasis, which are the substantial causes of therapeutic failures.

Moreover, since the efficacy of anti-cancer treatments is affected by the level of ncRNAs [21,22,277], the combination of anti-cancer drugs with ncRNA-based therapy can further improve the therapeutic responses. For example, the effect of simvastatin on the repression of cell survival and metastasis is enhanced by the combined inhibition of miR-21 [278]. Also, miR-126-3p reconstitution synergistically improves the combinatorial effects of PIK-75 with vemurafenib on apoptotic cell death in vivo [279]. In addition, lncRNA-TCL6 overexpression can potentiate the rate of apoptotic cell death induced by paclitaxel [280]. Further investigations into the combinatorial effects of anti-cancer drugs with ncRNA-based therapy on anoikis, anchorage-independent growth, and metastasis are warranted.

Accumulating evidence has shown that extracellular vesicles (EVs), such as exosomes and microvesicles, can deliver ncRNAs and mediate mutual communication between heterogeneous cell populations in the cancer microenvironment [281,282,283]. Cargo molecules in EVs exert influence on cancer aggressiveness. For example, miR-10a-5p and lncRNA-SOX2 overlapping transcript (lncRNA-SOX2OT) are cargo molecules in EVs derived from cancer-associated fibroblasts (CAFs) and invasive cancer cells, respectively, enhancing the metastatic capacity of circumjacent cancer cells [284,285]. In addition, there are only a few studies that demonstrate the function of ncRNA cargo in association with anoikis resistance and anchorage-independent cell growth. EVs secreted from gemcitabine-resistant cells contain miR-222-3p, and this miRNA confers anoikis resistance in EV-receiving cells [286]. Recently, it was demonstrated that miRNAs, such as miR-21, miR-143, and miR-378e, are abundant in EVs from CAFs and are able to increase anchorage-independent cell growth [287]. Further investigations are necessary to clarify the role of ncRNA cargo molecules in regulating anoikis resistance and anchorage-independent cell growth, the hallmarks of metastatic cancer cells. Moreover, the alteration of immune cell function can lead to failure in the elimination of metastatic cancer cells, promoting the development of metastasis [288]. Accumulating evidence demonstrated that ncRNA cargo in EVs can modulate the function of immune cells within the cancer microenvironment and that reciprocal communication between cancer cells and immune cells can affect cancer metastasis. For example, exosomal miR-301a from hypoxic cancer cells supports the development of M2-type tumor-associated macrophages, which serve as metastasis promoters in the cancer microenvironment [289,290]. It was also demonstrated that miR-203 in cancer cell-derived exosomes is delivered into dendritic cells (DCs). This miRNA reduces the level of cytokines involved in the maturation of DCs, suggesting that miR-203 can weaken the anti-cancer responses of T cells via inactivating DCs [291]. Overall, these findings suggest that ncRNA cargo molecules can be beneficial targets for cancer metastasis.

For therapeutic purposes, however, the proper selection of ncRNAs is particularly necessary depending on the cancer type owing to their dual roles, as stated in Section 2.1.2, Section 2.2.1, Section 4.6 and Section 4.7. Moreover, it should be concerned about functional dissimilarities between miR-3p and miR-5p for vector-based miRNA therapies. For instance, in contrast to miR-520f-5p that potentiates anchorage-independent cell growth (see Section 2.1.5), miR-520f-3p derived from the identical precursor of miR-520f-5p was reported to act as a tumor-suppressive miRNA by inactivating Wnt/β-catenin signaling [292]. Advanced knowledge of the cancer-related functions and characteristics of ncRNAs will enable the establishment of clinically relevant strategies for ncRNA-based cancer therapy.

## Figures and Tables

**Table 1:** Oncogenic miRNAs that enhance the anoikis resistance and anchorage-independent growth of cancer.

miRNA	Type of Cancer	In Vitro Experiment	In Vivo Experiment	Ref.
miR-9-5p	Hepatocellular carcinoma	Plating cells on polyHEMA-coated plates + annexin-V/7-AAD analysis *	Subcutaneous injection of SK-Hep1 cells and Huh7 stably overexpressing and knocking down GALNT4, respectively	[29]
miR-10b	Melanoma	Soft agar assay **	-	[30]
miR-21-5p	Esophageal cancer	Plating cells on polyHEMA-coated plates + annexin-V/propidium iodide analysis *	Tail vein injection of OE33 cells stably overexpressing or knocking down miR-21	[27]
miR-25-3p	Retinoblastoma	Soft agar assay **	Subcutaneous injection of WERI-RB-1 cells transduced with miR-25-3p lentiviral plasmids	[28]
miR-27-3p	Osteosarcoma	Soft agar assay **	-	[31]
miR-106-5p	Pituitary adenoma	Agarose clonal formation assay **	-	[32]
miR-139-5p	Adrenocortical cancer	Soft agar colony formation assay **	-	[33]
miR-141-3p	Ovarian cancer	Plating cells on 1% agarose gel coated plates + TUNEL assay *	Intraperitoneal injection of SKOV3 cells stably expressing miR-141-3p	[34]
miR-141-3p	Bladder cancer	Soft agar assay **	Subcutaneous injection of TAp63α-expressing UMUC3 cells	[35]
miR-145-5p	Esophageal cancer	Plating cells on low adhesion plates + Western blotting for detecting PARP cleavage *	Flank injection of SK-GT-4 cells overexpressing miR-145	[36]
miR-145-5p	Bladder cancer	Soft agar assay **	Subcutaneous injection of T24T cells stably overexpressing miR-145-5p	[37]
miR-146-3p	Cervical cancer	Soft agar assay **	-	[38]
miR-182-5p	Bladder cancer	Soft agar colony formation assay **	-	[39]
miR-186-5p	Prostate cancer	Soft agar assay **	-	[40]
miR-186-5p	Melanoma	Soft agar assay **	-	[41]
miR-206	Breast cancer	Cell culture using 1% methylcellulose-based media + propidium iodide staining/trypan blue exclusion *	Orthotopic injection of MDA-MB-231 cells treated with miR-206 inhibitors	[42]
miR-362-3p	Hepatocellular carcinoma	Soft agar colony formation assay **	-	[43]
miR-376c-3p	Gastric cancer	Soft agar assay **	-	[44]
miR-411-5p	Hepatocellular carcinoma	Soft agar assay **	-	[45]
miR-421	Lung cancer	Soft agar assay **	-	[46]
miR-424-5p	Thyroid cancer	Plating cells on polyHEMA-coated plates + annexin-V/propidium iodide analysis *	Tail vein injection of K1 cells stably overexpressing or knocking down miR-424-5p	[47]
miR-483-5p	Adrenocortical cancer	Soft agar colony formation assay **	-	[33]
miR-494	Colorectal cancer	Soft agar assay **	-	[48]
miR-520f-5p	Melanoma	Soft agar assay **	-	[49]
miR-526b-5p	Breast cancer	Spheroid formation assay **	Intravenous or subcutaneous injection of CPEB2A-silencing MCF10A cells	[50]
miR-527	Esophageal cancer	Soft agar assay **	-	[51]
miR-582-5p	Colorectal cancer	Soft agar assay **	-	[52]
miR-645	Colorectal cancer	Soft agar assay **	Subcutaneous injection of WiDr and EB cells stably knocking down miR-645	[53]
miR-655-3p	Breast cancer	Spheroid formation assay **	Intravenous or subcutaneous injection of CPEB2A-silencing MCF10A cells	[50]
miR-760	Ovarian cancer	Soft agar assay **	-	[54]
miR-766-5p	Colorectal cancer	Soft agar assay **	-	[55]
miR-1288-3p	Glioblastoma	Soft agar colony formation assay **	-	[56]
miR-1303	Neuroblastoma	Soft agar assay **	-	[57]
miR-3607	Lung cancer	Soft agar assay **	-	[58]
miR-G-10	Cervical cancer	Cell–matrix adhesion assay **	Intravenous injection of HeLa cells expressing miR-G-10	[59]

* anoikis assays. ** assays for the measurement of anchorage-independent growth.

**Table 3:** LncRNAs that affect the anoikis resistance and anchorage-independent cell growth in cancer.

LncRNA	Type of Cancer (Expression of lncRNA)	In Vitro Experiment	In Vivo Experiment	Ref.
LncRNA-ANRIL	Glioblastoma (↑)	Plating cells on polyHEMA-coated plates + annexin-V assay *	-	[242]
LncRNA-FOXD2-AS1	Thyroid cancer (↑)	Soft agar assay **	Subcutaneous injection of K1 cells stably silencing lncRNA-FOXD2-AS1	[243]
LncRNA-H19	Gastric cancer (↑)	Soft agar assay **	-	[244]
LncRNA-HOTAIR	Gastric cancer (↑)	Soft agar assay **	Tail vein injection of MKN74 cells expressing LncRNA-HOTAIR. Intraperitoneal injection of KATO III cells following lncRNA-HOTAIR knockdown	[245]
LncRNA-HOTAIR	Gastric cancer (↑)	Plating cells on ultra-low attachment plates + annexin-V and MTT assay *	Intraperitoneal injection of MKN45 cells following the transfection of lncRNA-HOTAIR-targeting siRNA	[246]
LncRNA-LEF1-AS1	Colorectal cancer (↑)	Soft agar assay **	Tail vein injection of HT29 cells following the knockdown of lncRNA-LEF1-AS1	[247]
LncRNA-MEG3	Hepatocellular carcinoma (↓)	Soft agar assay **	-	[248]
LncRNA-NEAT1	Cervical cancer (↑)	Soft agar assay **	-	[249]
LncRNA-SNHG12	Renal cell carcinoma (↑)	Soft agar assay **	Subcutaneous injection of Caki-1 cells stably silencing lncRNA-SNHG12	[250]
LncRNA-TINCR	Breast cancer (↑)	Soft agar assay **	Subcutaneous injection of MDA-MB-468 cells following the transfection of lncRNA-TINCR-targeting siRNA	[251]

* anoikis assays. ** assays for the measurement of anchorage-independent growth.
